# The old age health security in rural China: where to go?

**DOI:** 10.1186/s12939-015-0224-5

**Published:** 2015-11-04

**Authors:** Baozhen Dai

**Affiliations:** Department of Health Policy and Management, School of Management, Jiangsu University, 301 Xuefu Road, Zhenjiang, 212013 China; Department of Health Policy and Management, Bloomberg School of Public Health, Johns Hopkins University, 624 North Broadway, Baltimore, 21205 USA

**Keywords:** China, Rural, Elder, Old age health security, New cooperative medical scheme, Social medical assistance scheme

## Abstract

**Introduction:**

The huge number of rural elders and the deepening health problems (e.g. growing threats of infectious diseases and chronic diseases etc.) place enormous pressure on old age health security in rural China. This study aims to provide information for policy-makers to develop effective measures for promoting rural elders’ health care service access by examining the current developments and challenges confronted by the old age health security in rural China.

**Methods:**

Search resources are electronic databases, web pages of the National Bureau of Statistics of China and the National Health and Family Planning Commission of China on the internet, China Population and Employment Statistics Yearbook, China Civil Affairs’ Statistical Yearbook and China Health Statistics Yearbooks etc. Articles were identified from Elsevier, Wiley, EBSCO, EMBASE, PubMed, SCI Expanded, ProQuest, and National Knowledge Infrastructure of China (CNKI) which is the most informative database in Chinese. Search terms were “rural”, “China”, “health security”, “cooperative medical scheme”, “social medical assistance”, “medical insurance” or “community based medical insurance”, “old”, or “elder”, “elderly”, or “aged”, “aging”. Google scholar was searched with the same combination of keywords.

**Results:**

The results showed that old age health security in rural China had expanded to all rural elders and substantially improved health care service utilization among rural elders. Increasing chronic disease prevalence rates, pressing public health issues, inefficient rural health care service provision system and lack of sufficient financing challenged the old age health security in rural China.

**Conclusions:**

Increasing funds from the central and regional governments for old age health security in rural China will contribute to reducing urban–rural disparities in provision of old age health security and increasing health equity among rural elders between different regions. Meanwhile, initiating provider payment reform may contribute to improving the efficiency of rural health care service provision system and promoting health care service access among rural elders.

## Background

By the end of 2014, the number of elders (aged 65 years old and above) had reached 138 million in China, accounting for 10.1 % of the total population [1]. Moreover, it was estimated that the number of elders may increase to 450 million (nearly one third of the total population) by 2050 [2]. Nearly 60 % elders live in rural areas [3]. With the number of young adults migrating to urban areas increased rapidly in recent decades, elders become the primary residents remaining in rural areas [4]. The huge number of rural elders places enormous pressure on old age health security in rural China [5].

The old age health security in rural China, which aims to promote rural elders’ health status and prevent poverty caused by illness, includes social health insurance scheme (new cooperative medical scheme, NCMS) and social medical assistance scheme. In 2003, the Ministry of Health of the People’s Republic of China launched NCMS in rural areas, and the Ministry of Civil Affairs of the People’s Republic of China launched social medical assistance scheme for those rural residents with low-income. For those rural elders who are poverty-stricken, the rural social medical assistance scheme will subsidize them to participate in NCMS or proportionally reimburse them when they can not afford out-of-pocket payments of medical expenses [6].

Studies showed that NCMS and the social medical assistance scheme have had substantial impact on improving rural elders’ health care service utilization [7–9], and the general health and psychological health among rural elders have been improved [10]. However, the improvements in rural elders’ health care service access is limited due to the deepening health problems in rural areas brought by the process of China’s transformation towards an industrialized society, such as huge disparities in health care service provision between urban and rural areas, health inequity between different rural areas, serious environmental degradation and growing threats of infectious diseases and chronic diseases [11–14].

There are few studies on the current developments and challenges confronted by the old age health security in rural China, and little information is available for policy-makers to further improve the old age health security in rural China. This study aims to provide information for policy-makers to develop effective measures for promoting rural elders’ health care service access by examining the current developments and challenges confronted by the old age health security in rural China.

## Methods

The literature selection was conducted during November 6, 2011 to May 20, 2012. Search resources are electronic databases, web pages of the National Bureau of Statistics of China and the National Health and Family Planning Commission of China on the internet, China Population and Employment Statistics Yearbook, China Civil Affairs’ Statistical Yearbook and China Health Statistics Yearbooks etc.

The databases searched were Elsevier, Wiley, EBSCO, EMBASE, PubMed, SCI Expanded, ProQuest, and National Knowledge Infrastructure of China (CNKI) which is the most informative database in Chinese. Search terms were “rural”, “China”, “health security”, “cooperative medical scheme”, “social medical assistance”, “medical insurance” or “community based medical insurance”, “old”, or “elder”, “elderly”, or “aged”, “aging”. Google scholar was searched with the same combination of keywords. Selected papers were numbered and the reference lists were reviewed with attention paid to the duplicates. Refined searches were performed based on the following inclusion criteria: 1) written in English or Chinese; 2) about rural China; 3) published after 1949; 4) is a reviewed article, non-reviewed report, or government documents. There are, in total, 31 papers selected, including 7 reviews.

## Results

### Developments of old age health security in rural China

#### Social health insurance scheme for rural elders

Social health insurance scheme in rural China is one of the key components of rural old age health security. It plays an important role in guaranteeing rural elders’ access to the basic and necessary health care services. The cooperative medical system (CMS), which was the rural social health insurance scheme, was set up in the 1950s. It was financed by rural collective economy and provided health care services by a three-tiered health care service provision system which includes village clinics, township health care service centers and county hospitals [15–17]. Before the rural economic reform initiated in the late 1970s, rural residents were covered by CMS, and most medical expenses of health care services among rural residents were paid by CMS.

After the rural economic reform, CMS collapsed gradually and rural residents had to pay for themselves when ill. In 2003, there were about 80 % rural residents not covered by social health insurance scheme [18]. Meanwhile, since the late 1970s, the government of China didn’t finance rural health care provision system sufficiently, and hospitals had to support themselves economically. For profit, hospitals ran into a disordered competition, leading to a sharp decline of the number of township health care center and village clinic from the late of 1970s through the 1990s [19]. Medical treatment received unprecedented attention for its much higher profits than those of preventive health care services. Public health, which played an important role in providing preventive health care services, was ignored in rural areas. Without covering by CMS, rural residents’ out-of-pocket payments increased by 106 % (of rural resident’s net income per capita) in 1991 to 149 % (of rural resident’s net income per capita) in 2003, and about 30 % rural families were impoverished by high medical expenses [20, 21]. For rural elders, of whom 66.7 % had middle and low incomes, health care services became less accessible to them than ever [22].

In 2003, the government of China launched new cooperative medical scheme (NCMS). By the end of 2013, NCMS has expanded to 2489 counties, covering 802 million rural residents (accounting for 98.7 % of the total rural population) [23]. NCMS funds are raised from the central government, regional government, local government, village committees and the participants, with rural residents’ voluntary enrollment. The average amount of funds of NCMS per capita increased from 30 RMB in 2003 to 380 RMB in 2015 [24]. The risk pool of NCMS is established and managed at county-level, and county government is authorized to design and management NCMS according to the local social-economic development situation [25]. Till now, there are dramatic variations in NCMS design and management between counties, even in the same province.

Studies showed that NCMS improved rural residents’ health care service utilization with an increase in outpatient visits by 23 % and inpatient episodes by 27 % [7, 26]. Special considerations of NCMS were encouraged to be given to elders in rural areas, such as premiums remission and free check-up. Rural elders, who had less access to health care services before the initiation of NCMS, showed the highest satisfaction with NCMS among all age groups [27, 28]. However, among those rural elders who had participated in NCMS, more than a half still faced affordability difficulty when ill, and the number of rural elders whose needs for hospitalization were unmet accounted for 18.25 % of the total rural elders [29]. Some impoverished rural elders with poor physical health and functional limitations may lack sufficient access to basic and necessary health care services due to their poor affordability [7].

#### Social medical assistance scheme for rural elders

Social medical assistance scheme in rural China was initiated by the government of China in 2003. The funds of rural social medical assistance scheme are raised from the governments and social donation [6]. For those *wubao* elders who have lost their abilities and income to maintain a normal life and those who are too poor to afford the premiums of NCMS, rural social medical assistance scheme will pay NCMS premium for them. For those rural elders who have participated in NCMS, rural social medical assistance scheme will proportionally reimburse them when they suffer form catastrophic out-of-pocket payments of medical expenses.

In 2005, social medical assistance scheme was required to expand to all rural areas and cover those elders who were *wubao* and those with a minimum living allowance at least [30]. From 2004 to 2008, the number of rural residents who had been financed by rural social medical assistance scheme increased from 6.74 million to 41.92 million, the number of those who had been paid for NCMS premium by rural social medical assistance scheme increased from 5.53 million to 34.32 million, and the number of those who had been proportionally reimbursed with out-of-pocket payments increased from 1.21 million to 7.60 million [31].

In 2009, social medical assistance scheme in rural China was required to cover all rural low-income families with catastrophic medical expenses as well as those poverty-stricken residents defined by local governments including *wubao* elders and those with a minimum living allowance [32]. From 2009 to 2011, the number of rural residents who had been financed by rural social medical assistance scheme increased from 47.89 million to 62.97 million, the number of those who had been paid for NCMS premium increased from 40.59 million to 48.25 million, and the number of those who had been proportionally reimbursed with out-of-pocket payments increased from 7.30 million to 14.72 million. In 2011, the average level of spending for NCMS premium was 45.6 RMB per capita, and the average level of spending for rural residents’ out-of-pocket payments was 635.8 RMB per capita [31].

### Challenges confronted by old age security in rural China

The increasing social economic disparities between rural and urban areas have caused a huge migration from rural to urban areas since the late 1990s. In 2014, there were 274 million rural labors migrating to urban areas, and the number of those who migrated with their wives and children increased from 28.6 million in 2008 to 35.8 million in 2014 [33].With the number of young labors who migrated to urban areas with their wives and children increased rapidly, rural elders became the main groups left behind in rural areas.

In recent decades, rural areas has born most of the negative environmental impacts brought by industrialization transformation, and the environmental degradation has led to an increase of chronic disease prevalence rates among rural elders [11, 12, 34, 35]. Although studies showed that the chronic disease rates of the urban elderly were higher than those of their counterparts in the rural, risk exposure was more common and community health services were less effective in rural areas, and apparent better health of rural elderly might be partly explained by under-diagnosis, under-reporting, or selective mortality in view of their limited health care service access [14].

Though China has achieved great success against tuberculosis and schistosomiasis since 1949, public health issues are still prominent in rural areas [36]. Since the economic reform in the late 1970s, the provision of public health diminished quickly in rural areas and the risks of infectious disease transmission increased rapidly with the allocation of public funds heavily skewed towards urban areas [37]. In recent decades, infectious diseases (e.g. tuberculosis and schistosomiasis) are resurgent in rural China, and the threats of HIV/AIDS and Avian Flu are growing. Meanwhile, mental health problem became an important issue among rural elders in recent decades. Studies showed that suicide rates among rural residents were three times higher than those of their urban counterparts, and the highest suicide rates were among rural elders’ (aged 60 years old and above) within all age groups in rural areas [36].

Compared to the urban health care service provision system which had been improved greatly since 1978, the rural three-tiered health care service provision system (village clinics, township health care service centers, and county hospitals) had experienced the collapse of CMS which was the financing system of rural health care service provision system and the sharp reduction in the number of “barefoot doctors” who are the main health care service providers in village clinics [38]. Since the late 1970s, public health in rural areas was left behind due to financial constraints, and reduction in public health campaigns and health awareness initiatives had an undesirable side-effect on the rural health care service provision system, resulting in inadequate safeguards and regulation of disease surveillance [36]. Meanwhile, health care service provision system for non-communicable diseases was insufficient in rural areas with less qualified of staff and low quality of services [39]. Due to the major financing responsibility shifting to local governments, an unevenly distributed health care service provision capacity in rural areas exists among provinces, leading to inequity in health outcome among rural residents.

## Discussion

In recent decades, rural areas were socially and economically left behind in the process of China’s transformation towards an industrialized society [13]. The disparity between urban and rural areas has caused an unevenly distributed provision of old age health security between the urban and rural areas. For example, in 2011, the average level of spending for premium was 45.6 RMB per capita in rural areas while it was 67.9 RMB in urban areas, and the average level of spending for out-of-pocket payments was 635.8 RMB per capita in rural areas while it was 793.6 RMB in urban areas [31].

Meanwhile, as local governments take the major financing responsibility of old age health security, the difference in financial capabilities between local governments has led to rural elders’ health care service access varying greatly in different regions [25]. In social-economic under-developed rural areas, rural elders’ health care service access was very limited. Increasing funds from the central and regional governments for old age health security in rural China will contribute to reducing the urban–rural disparities in provision of old age health security and increasing health equity among rural elders between different regions.

Though the old age health security substantially improved health care service utilization among rural elders, affordable difficulties were still a common reason for rural elders not to seek health care services [40]. It has been reported that NCMS has not reduced out-of-pocket payments per outpatient visit or inpatient spell, and reimbursements of NCMS reduced catastrophic spending from 8.98 % of the households to 8.25 % with the incidence of catastrophic spending increasing deciles 3–10 [41, 42]. Moreover, NCMS participants with high income benefited more from NCMS than those with middle and low income [26]. Rural elders with middle and low income had limited access to health care services, and they were less likely to seek medical treatments when ill than other age groups of rural residents [3].

Additionally, inefficiencies in rural health care service provision system contribute a lot to the rapid growth in health expenditure and limited health care service access among rural elders. Fee-for-service (FFS), which is a payment pattern where health care services are paid for as itemized in the hospitals invoice, is adopted by most rural health care facilities since the collapse of CMS in the late 1970s. Evidence has been found that physicians paid under a FFS pattern tend to treat patients with more procedures than those paid under capitation or a salary [43]. In rural China, FFS has been proved to have a knock-on effect of an overprovision of unnecessary services and overuse of treatments and drugs [44]. Due to FFS depending on the quantity of health care service rather than the quality of health care service, hospitals become to encourage health care service providers to provide more health care services than ever for profits. Meanwhile, information asymmetry occurs between patients and health care service providers. With an illiteracy rate of 58.0 % among rural elderly, this informational disparity may be more severe between rural elderly patients and health care service providers than other age groups of rural residents [4]. With FFS and information asymmetry, health care service providers have a strong incentive to induce their patients to use clinically unnecessary services to increase their income [45]. Though NCMS was designed to promote health care service access among rural residents, evidence has been found that NCMS increased the utilization of expensive care and catastrophic out-of-pocket payments among rural residents [46, 47]. For old age health security in rural China, initiating provider payment reform may contribute to improving the efficiency of rural health care service provision system and promoting health care service access among rural elders.

## Conclusions

The old age health security in rural China has expanded to all rural elders and substantially improved health care utilization among rural elders. With rural elders being the main age group left behind in rural areas, the increasing chronic disease prevalence rates, pressing public health issues, inefficient rural health care service provision system and lack of sufficient financing challenged the old age health security in rural China. Increasing funds from the central and regional governments for old age health security in rural China will contribute to reducing urban–rural disparities in provision of old age health security and increasing health equity among rural elders between different regions. Meanwhile, initiating provider payment reform may contribute to improving the efficiency of rural health care service provision system and promoting health care service access among rural elders.
